# From positive screen to engagement in treatment: a preliminary study of the impact of a new model of care for prisoners with serious mental illness

**DOI:** 10.1186/s12888-016-0711-2

**Published:** 2016-01-15

**Authors:** Krishna Pillai, Paul Rouse, Brian McKenna, Jeremy Skipworth, James Cavney, Rees Tapsell, Alexander Simpson, Dominic Madell

**Affiliations:** Auckland Regional Forensic Psychiatry Services, Waitemata District Health Board, Auckland, New Zealand; The University of Auckland, Auckland, New Zealand; Australian Catholic University and NorthWestern Mental Health, Level 1 North, City Campus, The Royal Melbourne Hospital, Grattan Street, Parkville, VIC Australia 3050; Midlands Regional Forensic Psychiatric Service, Waikato District Health Board, Hamilton, New Zealand; Centre for Addiction and Mental Health, University of Toronto, Toronto, Canada

**Keywords:** Offender, Prisoner mental health, Mental health in-reach services, Mental health screening tool

## Abstract

**Background:**

The high prevalence of serious mental illness (SMI) in prisons remains a challenge for mental health services. Many prisoners with SMI do not receive care. Screening tools have been developed but better detection has not translated to higher rates of treatment. In New Zealand a Prison Model of Care (PMOC) was developed by forensic mental health and correctional services to address this challenge. The PMOC broadened triggers for referrals to mental health teams. Referrals were triaged by mental health nurses leading to multidisciplinary team assessment within specified timeframes. This pathway for screening, referral and assessment was introduced within existing resources.

**Method:**

The PMOC was implemented across four prisons. An AB research design was used to explore the extent to which mentally ill prisoners were referred to and accepted by prison in-reach mental health teams and to determine the proportion of prison population receiving specialist mental health care.

**Results:**

The number of prisoners in the study in the year before the PMOC (*n* = 19,349) was similar to the year after (*n* = 19,421). 24.6 % of prisoners were screened as per the PMOC in the post period. Referrals increased from 491 to 734 in the post period (Z = −7.23, *p* < 0.0001). A greater number of triage assessments occurred after the introduction of the PMOC (pre = 458; post = 613, Z = 4.74, *p* < 0.0001) leading to a significant increase in the numbers accepted onto in-reach caseloads (pre = 338; post = 426, Z = 3.16, *p* < 0.01). Numbers of triage assessments completed within specified time frames showed no statistically significant difference before or after implementation. The proportion of prison population on in-reach caseloads increased from 5.6 % in the pre period to 7.0 % in the year post implementation while diagnostic patterns did not change, indicating more prisoners with SMI were identified and engaged in treatment.

**Conclusions:**

The PMOC led to increased prisoner numbers across screening, referral, treatment and engagement. Gains were achieved without extra resources by consistent processes and improved clarity of professional roles and tasks. The PMOC described a more effective pathway to specialist care for people with SMI entering prison.

## Background

There is growing recognition of the plight of prisoners with serious mental illness (lifetime diagnosis of schizophrenia or bipolar disorder or current diagnosis of severe major depression; SMI). A recent meta-regression analysis of 33,588 prisoners in studies from 24 countries puts the pooled prevalence rates of SMI at 13.8 % of male prisoners and 18 % for female prisoners [1]. The detection of prisoners with SMI is of paramount importance because through detection such prisoners can promptly access services for assessment and treatment; be supported through the legal process; be assisted with the processes of adjustment to incarceration; and be assisted with eventual community reintegration.

Yet research suggests that the majority of prisoners with SMI remain undetected and untreated. A study in the United Kingdom found that 23 % of prisoners surveyed in six prisons had a current diagnosis of SMI but only 25 % of the prisoners with SMI were assessed by in-reach mental health teams and only 13 % were accepted for treatment [2]. In New Zealand, only 37 % of prisoners identified as suffering from schizophrenia reported being under any form of treatment [3].

Recognising that early identification is the first step to the provision of treatment, considerable rigor has been applied to the development of screening tools for mental illness at reception into custody [4–6]. But detection by screening processes is only the first step in a referral process to access treatment for SMI. A study conducted in 5 English prisons, found more than 60 % of prisoners identified at the point of reception as taking psychotropic medication never received a mental health assessment and only 36 % received any medication in prison [7]. Similarly a review of screening processes in an urban assessment prison in Australia found that despite one fifth of new prison receptions being detected with serious mental health problems, less than 1 % were transferred to the areas of the prison where intensive mental health assessment and treatment were possible [8].

A participatory action research study of 20,084 consecutive male remands in Ireland [9] yielded 572 successful diversions of mentally ill prisoners from the criminal justice system to therapeutic settings over a 6 year period. This was achieved with substantial and sustained investment of resources and the commitment of clinicians working collaboratively with key stake holders to develop a local solution.

These studies highlight the importance of systematic and collaborative approaches to care pathways for prisoners with serious mental illness that include not only screening, but prescribed responses to positive screens which enable appropriate referral, assessment and sustained clinical engagement.

In New Zealand, mental health services for remand and sentenced prisoners are provided by forensic mental health teams in an in-reach model working alongside correctional primary health staff. We estimate that in-reach team caseloads should be 10 to 15 % of the standing prison population [10], consistent with the prevalence rates of SMI in pooled prison population studies [1]. In 2005, a national census found only 5.1 % of the nation’s prison population under mental health care [11]. Successive investments of public money created the platform for a more comprehensive approach to in-reach mental health treatment. However no overarching strategy for in-reach services existed.

In 2011, a Prison Model of Care (PMOC) was developed as an inter-regional initiative to improve the consistency and quality of prison mental health in-reach care in the northern part of New Zealand [12]. The PMOC divided the care pathway into five steps (screening, referral, assessment, treatment and release planning) of which the first three are important in the detection of prisoners with SMI. Prior to the PMOC no consistent evidence-based approach to mental health screening was occurring at the point of entry to prisons and the prison medical officer was the sole source of referral. There were no accepted standards for the rapidity of response.

**Fig. 1:**
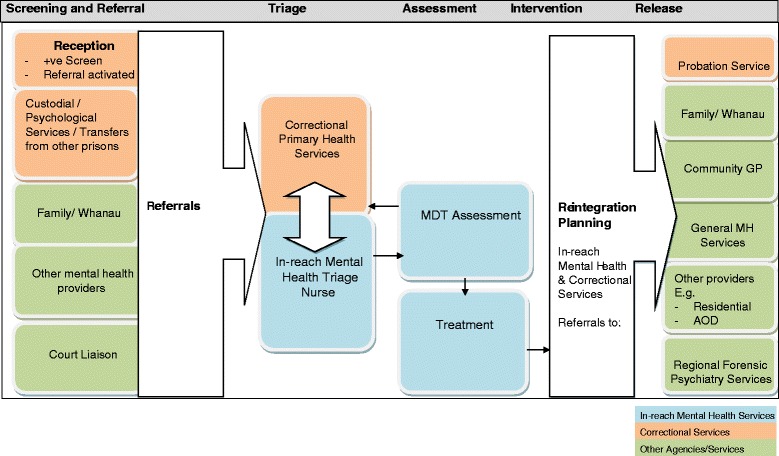
The Prison Model of Care Referral and Treatment Pathway

The PMOC requires screening at reception to prison be undertaken by prison primary health staff. The screening tool employed was a combination of the Brief Jail Mental Health Screen [13] and the English Mental Health Screen [4] and was validated with New Zealand male prisoners [5]. Figure 1 depicts the pathway commencing with screening, to referral, assessment, treatment and release. Screening is performed by the correctional primary health staff who refer positive screens to the prison in-reach mental health services for triage by a mental health nurse. Thus, in the PMOC, referrals to prison in-reach mental health services are triggered by a positive screen or by concerns raised from other sources including family, court liaison staff, prison officers and primary healthcare providers. Referrals are classified to be seen within 24 h, 72 h or within one week depending on acuity. Referrals lead to a triage interview by an in-reach mental health nurse and then to a full psychiatric assessment if deemed necessary. As shown in Fig. 1, referrals are either retained within the in-reach service for MDT assessment or are transferred back to the correctional primary health services. The PMOC requires collaborative working between correctional health services and prison in-reach mental health services.

There were no new resources allocated to bring about this change in service model. However, the implementation of the model was fully supported by the health and correctional services involved. Extensive briefings and information resources were provided to all clinicians in the prison in-reach teams. The implementation of the model was staggered at approximately three month intervals across different prison sites to allow time for liaison with prison authorities, alteration of work practices and reallocation of staff to support changing roles.

The aim of this paper is to describe the impact of the screening, referral and assessment pathway implementation on the proportion of prisoners receiving specialist mental health services. It was hypothesised that the structured pathway, building on the study of Evans et al. [5] would lead to improved screening, referral and assessment processes for prisoners with SMI and consequently increased caseloads for in-reach mental health teams. An investigation into the impact of this model on treatment and release planning for those prisoners is described elsewhere [12].

## Method

An AB research design was used to explore the extent to which mentally ill prisoners were referred to and accepted by prison in-reach mental health teams and to determine the proportion of the prison population receiving specialist mental health care. All prisoners in the four prisons at the start of the study period and prisoners newly received over the subsequent 12 months were included in the analysis. Key outcomes measured included (i) the proportion of new receptions receiving routine mental health screening (ii) the proportion of the prison population receiving specialist mental health care (iii) the proportion of triage assessments completed by mental health nurses and (iv) preservation of existing levels of clinical care as measured by the assessment of prisoners in accordance with their allocated priority times and maintenance of focus on targeted serious mental illness. These outcomes were compared in the year before and year after the implementation of the new screening, assessment and referral pathway and are reported in the results section.

The PMOC was established in four prisons [11]. These were: a remand prison for 966 male prisoners; and three minimum to maximum security prisons for 1979 male mixed remand and sentenced prisoners. All prisoners received into the prisons for the first time (new receptions) have a prison primary nursing assessment at the point of arrival in the institution. The combined BJMHS/EMHS tool was added to this nursing assessment as part of the PMOC initiative. Prisoners screening positive at reception, or prisoners for whom concerns were expressed that they might have a SMI, were referred to the in-reach mental team by correctional health staff. In-reach team mental health nurses then performed an initial triage assessment to determine whether the prisoner should be referred for further and more comprehensive mental health assessment. The comprehensive assessment led to a decision as to whether the in-reach team would provide further services to the prisoner. These are the processes referred to as the screening, referral, and assessment pathways (see Fig. 1).

Prisoners referred were identified through the electronic management systems in each of the prisons. Data were collected by electronic file review of the cases identified. All those in prison at the start of the pre and post implementation study periods and newly received over the subsequent 12 months were eligible for inclusion.

Subsequent to the completion of the study period, data regarding the proportion of the prison population on prison in-reach caseloads was collected. A trend analysis for three years after the completion of the study was undertaken to determine shifts in the percentage of prisoners with SMI on the caseload of the in-reach mental health team.

Data was transferred to Excel spreadsheets and analysed using descriptive statistics. The statistical significance of the results was tested using Z-tests.

The research was approved by the Upper South B Regional Ethics Committee (Ethics Ref: URB/10/12/053).

## Results

### Total number of prisoners and new receptions

The number of prisoners included in the study in the year before the implementation of the PMOC (*n* = 19349) was similar to the year after (*n* = 19421). In the studied prisons the proportion of new receptions to total prisoners was significantly greater in the year after the implementation of the PMOC than in the year before (Z = 8.91, *p* < 0.0001).

### Referrals to the mental health in-reach team

As noted, screening was conducted by the correctional primary care nurses as part of the implementation of this model. The timing of the implementation of the screening tool was managed by correctional services and was not under the control of the research team. Consequently only 24.6 % of new receptions were screened as per the model of care in the post implementation study period. Mental Health screening for new receptions became universal in the year after the post implementation study period.

Overall there were 491 referrals (2.5 % of those included in the study) in the pre period and 734 referrals (3.7 %) in the post period. The increase in referral numbers after the introduction of the PMOC was significant (Z = 7.23, *p* < 0.0001) (see Table 1). At the remand prison and the mixed prisons, there were significantly more referrals in the post-period than in the pre-period (remand prison Z = 3.23, *p* < 0.01; mixed prisons Z = 6.72, *p* < 0.0001).

**Table 1:** Number of prisoners at each detection stage

	Remand Prison		Mixed Prisons		Total	
	Pre	Post	Pre	Post	Pre	Post
Total prisoners in the study period^a^	8933	9578	10,416	9843	19,349	19,421
New receptions (% of total prisoners)	8527 (95.5)	9491 (99.1)	8502 (87.4)	8142 (82.7)	17,029 (88.0)	17,633 (90.8)
New receptions screened (% of new receptions)	0 (0)	1446 (15.2)	0 (0)	2893 (35.5)	0 (0)	4339 (24.6)
New referrals for triage assessment (% of total prisoners)	148 (1.6)	222 (2.3)	343 (3.3)	512 (5.2)	491 (2.5)	734 (3.7)
Triage assessments completed (% of referrals)	138 (93.2)	197 (88.7)	320 (93.3)	416 (81.3)	458 (93.3)	613 (83.5)
Triage assessments not completed (% of referrals)	10 (6.8)	25 (11.3)	23 (6.7)	96 (18.7)	33 (6.7)	121 (16.5)
Prisoners to MDT assessment and treatment (% of triage assessments)	108 (78.2)	147 (74.6)	230 (71.9)	279 (67.1)	338 (73.8)	426 (69.5)

^a^Prison population in first month plus subsequent new receptions over the next 12 months

### Triage assessment of referrals

Across all prisons the majority of referrals received triage assessments before and after the introduction of the PMOC (see Table 1). While a significantly greater number of triage assessments occurred after the introduction of the PMOC, (pre = 458 of 19,349, 2.4 %; post = 613 of 19,421, 3.2 %; Z = 4.74, *p* < 0.0001), there was also a greater proportion of those referred for whom a triage assessment was not completed (pre = 33 of 491, 6.7 %; post = 121 of 734, 16.5 %; Z = 5.50, *p* < 0.0001) (see Table 1). The most common reason for non-completion was prisoners being transferred or released before initial assessment could take place (see Table 2).

**Table 2:** Reasons for no triage assessment

	Pre	Post
Prisoner transferred or released	21	65
Prisoner too unwell	1	5
Prisoner declined to engage with team	1	33
Case accepted without triage	3	3
Prisoner suicide	1	0
Prisoner deemed inappropriate for service without initial assessment	3	9
Referral forwarded to another service	0	2
No reason recorded	2	3
Referral withdrawn	1	1
TOTAL	33	121

The proportion of triage assessments completed by mental health nurses increased from 336 of 458 (73.3 %) to 520 of 613 (84.8 %) (Z = 4.55, p < 0.0001) after implementation of the PMOC (remand prison, pre = 105 of 138, 76 %; post = 182 of 197, 92 %; Z = 4.191, *p* < 0.0001), (male sentenced prisons, pre = 231 of 320, 72 %; post = 338 of 416, 81 %; Z = 2.91, *p* = 0.004).

The pathway required triage assessments to be completed within certain time frames according to urgency assigned by the correctional primary care team. This data is presented in Table 3. Chi squared tests show no statistically significant difference in the completion of triage assessments within the categories of priority times between the pre and post period within the remand, mixed prisons and both combined.

**Table 3:** Completion of triage assessments within priority times

	Initial Assessment Completed within Time?	Remand		Mixed		Total	
		Pre	Post	Pre	Post	Pre	Post
24 h	Yes	3	1	22	23	25	24
	No	2	1	7	13	9	14
	Sub-total for 24 h	5	2	29	36	34	38
72 h	Yes	61	59	34	49	95	108
	No	47	54	24	30	71	84
	Sub-total for 72 h	108	113	58	79	166	192
1 week	Yes	24	66	178	252	202	318
	No	6	31	63	108	69	139
	Sub-total for 1 week	30	97	241	360	271	457
Total	Yes	88	126	234	324	322	450
	No	55	86	94	151	149	237
	Grand Total	143	212	328	475	471	687

### Total numbers of prisoners accepted onto in-reach mental health team caseloads

Overall there was a significant increase in the number of eligible prisoners detected and engaged with as new cases for comprehensive assessment and treatment by in-reach mental health teams after implementation (pre = 338 of 19349, 1.7 %; post = 426 of 19421, 2.2 %; Z = −3.16, *p* < 0.01).

### Proportion of prison population on caseload

The proportion of the combined prison populations on the in-reach caseload increased significantly between census dates chosen in the pre study period (1 June 2011) and the post period (1 June 2012) (pre = 161 of 2887, 5.6 %; post = 198 of 2825, 7.0 %; z = 2.18, *p* = 0.0295). Collation of data from subsequent census dates (see Table 4) after the completion of the formal study protocol shows a steady rise in this proportion to 9.8 % in the fourth year post implementation (2015).

**Table 4:** Proportion of prison population on mental health in-reach case load

Census Date	Mental Health In-Reach Case Load/Prison Population		
	Remand	Mixed Prisons	Total
29.6.10	35/810 (4.3 %)	112/1988 (5.6 %)	147/2798 (5.2 %)
1.6.11^a^	28/785 (3.6 %)	133/2102 (6.3 %)	161/2887 (5.6 %)
1.6.12^b^	36/953 (3.8 %)	162/1872 (8.6 %)	198/2825 (7.0 %)
1.5.13	49/908 (5.4 %)	184/1825 (10.0 %)	233/2733 (8.5 %)
27.5.14	69/963 (7.2 %)	187/1866 (10.0 %)	256/2829 (9.0 %)
24.5.15	93/976 (9.5 %)	182/1843 (9.9 %)	275/2819 (9.8 %)

^a^Within the pre PMOC study period for all four prisons

^b^Within the post PMOC study period for all four prisons

### Diagnostic mix of caseload

**Fig. 2:**
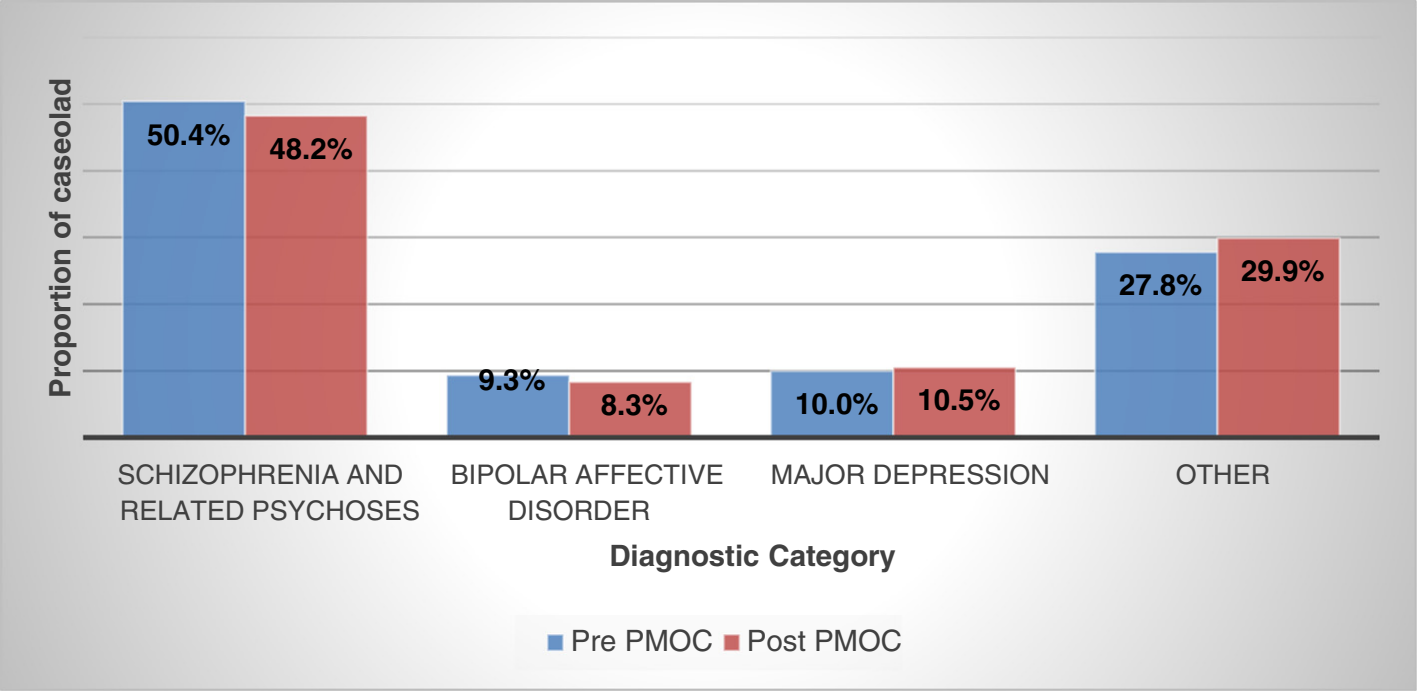
Diagnostic cumulative composition of in-reach case load under treatment over 12 month study period

Clinician recorded primary diagnosis of SMI from accumulated cases over 12 months showed little change between the pre and post implementation periods as depicted in Fig. 2. Just under one third of individuals in the pre and post study period attracted a diagnosis other than the targeted SMI. These findings are discussed below.

## Discussion

This is a descriptive study of the implementation of a screening, referral and assessment pathway (as part of a broader PMOC) for prisoners with SMI, in four prisons. This pathway required screening at reception to prison to be undertaken by correctional primary health care staff and also broadened the range of referral pathways to increase the likelihood that those missed by screening would be identified by another route. It is reasonable to predict better detection of SMI in prisons given such collaboration and improved rates of referral were evident in all prisons. Further the proportion of prisoners on specialist in-reach mental health team caseloads rose.

This was a naturalistic study in which not all variables impacting on outcomes could be manipulated. The evidence-based screening was implemented by the Department of Corrections partway through the post implementation study period so that only a quarter of prisoners accessed this part of the PMOC. The data collected relied on clinical file information collected for purposes other than research. Data collection for the post-PMOC period also commenced from the day of introduction, without giving the new ways of working time to become established. This likely reduced the size effect of the intervention.

Despite these limitations, this study identified modest improvements in key outcomes after the implementation of the screening, referral and assessment pathway. The initiative led to increased output across all stages of the pathway to mental health care. After implementation, broadened referral pathways and partly introduced screening processes led to more referrals. More referrals led to more triage assessments within specified time frames and more prisoners accepted onto the in-reach team caseload.

Efficiency gains (increased outputs within static resources) were achieved by reallocation of tasks. Mental health nurses were significantly more involved in triage assessments in the prisons after the introduction of the model of care, freeing medical officers and other team members to be more involved in other aspects of service delivery such as treatment and release planning described elsewhere [12].

Despite significant system changes, there was no difference between the diagnostic composition of the accumulated in-reach case load in the 12 months prior to and the 12 months after the implementation of the PMOC indicating that similar cases were being seen albeit in greater numbers. Furthermore a greater number of triage assessments were achieved without any significant changes in numbers seen within priority times. These two findings indicate that the new model was implemented without adverse impact on service provision. The consistent and sizeable proportion of the in-reach case load outside of the targeted diagnostic categories of SMI (pre = 27.8 %; post = 29.9 %) likely reflects the real-world clinical need to support and assist prisoners with a broad range of psychiatric disabilities (eg severe anxiety disorders, organic impairments, personality disorder) whose needs have been identified by this pathway.

Although there was delayed implementation of screening, the new process led to a significant rise in the proportion of the prison population on in-reach caseloads. This initially modest gain became more substantial over subsequent years and in the fourth year after implementation this key indicator reached almost 10 %. These observations should be treated with caution as they were not part of the original study design. Nonetheless there is an indication that there has been a gradual accumulation of cases under care as the evidence-based screening, expanded referral pathways and focus on collaborative case finding have been sustained and become standard ways of practicing. Repeating the diagnostic survey would be an important avenue for future quality measurement to ensure that a focus on SMI has been maintained.

Even with this approach caseloads have not reached the 15 % target population identified by epidemiologic research. As indicated in Table 2, a sizeable number of referred prisoners were released or transferred prior to the triage assessment highlighting the transient nature of this population, many of whom have short remand periods. Further there are likely some prisoners who never reach the point of screening. This raises the possibility that higher rates of detection may require screening that is even earlier in the criminal justice pathway and or triage assessment that is even more rapid.

This screening, referral and assessment pathway development has led to more prisoners with SMI receiving specialist treatment. This can be seen as a qualified success for evidence-based prison mental health service development, which moved from epidemiologic survey and screening tool development to the implementation of a systemic intervention yielding a positive outcome for prisoners with SMI.

## Conclusion

The PMOC lead to increased prisoner numbers across all stages of an in-reach mental health pathway from screening to referral to treatment and engagement. Gains were achieved without extra resources by consistent processes and improved clarity of professional roles and tasks. The screening, referral and assessment pathway from the PMOC describes a more effective pathway to specialist care for people with SMI entering prison. This sets the platform for needs based interventions to enable safe and successful community integration for this transient and high risk population.

## Abbreviations

AB research design: a two phase design consisting of a no-intervention baseline phase (A) and an intervention phase (B). It allows for evaluation of pre-intervention and intervention problem status
PMOC: prison model of care
SMI: serious mental illness
